# Retracted: Serum Fetuin-A Levels in Patients with Cardiovascular Disease: A Meta-Analysis

**DOI:** 10.1155/2020/2013691

**Published:** 2020-09-05

**Authors:** BioMed Research International

BioMed Research International has retracted the article titled “Serum Fetuin-A Levels in Patients with Cardiovascular Disease: A Meta-Analysis” [1]. This article is one of a series of very similar meta-analyses written by different authors in 2014, characterized by searching the complementary and alternative medicine database CISCOM despite the topic not being about CAM [2]. The articles have the same structure, with the figures in the same order. The appearance of the figures and parts of the text are also similar.

The study reports significant publication bias and this is consistent with the funnel plot, but there is no analysis or discussion of how this might have affected the results. Fetuin-A genetic variants associate with cardiovascular disease, which is consistent with the finding of this meta-analysis that reduced serum levels of Fetuin-A are associated with increased cardiovascular risk [3].

The authors explained that they were interested in Fetuin-A and the corresponding author's students collected the data and wrote the meta-analysis. They provided the full list of excluded studies and the complete search strategy for each database, which are being made available as supplementary files[Supplementary-material supplementary-material-1]. However, the authors asked to retract the article. They said that after expanding their literature review, they found that the conclusion in the article was not rigorous.

## Supplementary Materials

Supplementary Materials The full list of excluded studies and the complete search strategy for each database.
