# Supplementary material for: Retracted: Serum Fetuin-A Levels in Patients with Cardiovascular Disease: A Meta-Analysis
Source: Biomed Res Int. 2020 Sep 5;2020:2013691. doi: 10.1155/2020/2013691 (PMC7648669; doi:10.1155/2020/2013691)
Supplement: Supplementary Materials — The full list of excluded studies and the complete search strategy for each database. [file 2013691.f1.zip › 4241_followup_691540.Details of who conducted the meta (Authors Attachment).docx]

**Question 1:** Details of who conducted this meta-analysis and when this was done

**Reply:** This meta-analysis was conducted by Qi-Ying Xie and Ze-Lin Sun and completed in June 2014.

**Question 2:** The full list of the excluded studies

**Reply:** The full list of the excluded studies has been included in txt and is named "full list of the excluded studies".

**Question 3:** The complete search strategy for each database they searched

**Reply:** The complete search strategy for each database has been placed in the file of "search strategy".

**Question 4:** An analysis of how publication bias may have affected the results of the meta - analysis

**Reply:** Publication bias may affect the results of the meta-analysis. The only solution is to collect all the data related to the current systematic evaluation as far as possible. However, in this paper, we have developed retrieval strategies for 9 databases and screened the literatures, so it is relatively comprehensive. Then from the sensitivity analysis, it was found that the arbitrary deletion of one of the literatures had no effect on the conclusion of this study, indicating that the results were very stable. Now a better method may still be to publish literatures with a larger sample size and increase the number of included literatures.

**Question 5:** A study protocol containing the plan of how the meta-analysis was going to be conducted, from before the study began.

**Reply:**

1. Topic selection: raise questions, determine research objectives and formulate research plans

2. Establish inclusion and exclusion criteria and conduct literature search

3. Preliminary literature selection

4. Quality evaluation of the included literature

5. Data information of the included literature was extracted

6. Make statistical analysis and forest map with software

7. Heterogeneity analysis

8. Analysis of publication bias

9. Result analysis and discussion

10. Follow the PRISMA to write the full text
